# Pretreatment Fatigue in Breast Cancer Patients: Comparison With Healthy Controls and Associations With Biopsychosocial Variables

**DOI:** 10.1002/cam4.70404

**Published:** 2025-01-09

**Authors:** Patricia Blickle, Martina E. Schmidt, Karen Steindorf

**Affiliations:** ^1^ Division of Physical Activity, Prevention and Cancer German Cancer Research Center (DKFZ) Heidelberg Germany; ^2^ Division of Physical Activity, Prevention and Cancer, National Center for Tumor Diseases (NCT) Heidelberg, a Partnership Between DKFZ and University Medical Center Heidelberg Germany; ^3^ Medical Faculty University of Heidelberg Heidelberg Germany

**Keywords:** breast cancer patients, cancer, cancer‐related fatigue, oncology, patient‐reported outcomes, pretreatment fatigue, psychological factors, psycho‐oncology, quality of life, supportive care

## Abstract

**Objective:**

Cancer‐related fatigue is one of the most common burdens of cancer patients. To date, most studies focused on fatigue during or after treatment. However, investigation of pretreatment fatigue is crucial to identify causal or risk factors other than cancer therapy and to enable timely fatigue management.

**Methods:**

Two hundred and thirty‐two breast cancer patients (mean age = 55.6) and 41 healthy participants (mean age = 49.3) were recruited via the National Center for Tumor Diseases (NCT) Heidelberg. Patient‐reported outcomes were assessed with the EORTC QLQ‐FA12 for fatigue, the EORTC QLQ‐C30 for functioning, the STAI for anxiety, the CESD‐R for depression and the PSQI for sleep disturbance. Descriptive analyses and logistic regression models were performed using baseline data before start of cancer treatment. The thresholds of clinical importance (TCI) were applied to test for clinically relevant fatigue.

**Results:**

Compared to the healthy participants, patients scored significantly higher in physical, emotional, and total fatigue, in depression, in global health status and in all functioning scales except cognitive function (all *p* < 0.01). 48.7% of all patients reported clinically relevant fatigue. Being younger, being obese, having low education, or low social support was associated with a higher likelihood of clinically relevant fatigue before treatment. Higher depression and anxiety scores, poorer sleep quality and global health status, and impaired functioning seemed to get along with an increased likelihood of scoring above the TCI of fatigue (all *p* < 0.001).

**Conclusions:**

Our study results suggest that fatigue screening, patient‐centered fatigue education and psychosocial support may be needed already from the time of cancer diagnosis.

## 1 Background

Cancer‐related fatigue (CRF) is one of the most prevalent and burdensome side effects of cancer and cancer treatments. It manifests as a persistent feeling of physical, emotional, or cognitive weariness or exhaustion. It exhibits a disproportionate intensity relative to recent activity while impeding patient's ability to function [1]. Fatigue is not resolved by sleep or rest. Beside the fact that fatigue can lead to significant decrements in patients' quality of life (QoL), it has also been associated with lower recurrence‐free and overall survival [2].

The pathogenesis of fatigue is complex and multifactorial. Several factors have been suggested to contribute to the development of fatigue: [3] the tumor itself, its treatment with chemotherapy, radiotherapy or immunotherapy and inflammatory processes, such as increased levels of inflammatory cytokines or cortisol dysregulation. Reduced energy metabolism or (neuro)endocrine changes can also cause fatigue. In addition to hormonal changes, an unbalanced diet and a lack of physical activity are discussed as possible influences on the development of fatigue. Psychological factors such as anxiety, depressive symptoms, sleep disturbance, and stress also appear to be important contributors to fatigue, although the extent and causal mechanisms are not fully understood [4, 5].

In the course of treatment, around 85% of patients experience fatigue, while approximately one third experiences fatigue for more than 5 years after their treatment [6, 7, 8]. Some studies showed that fatigue can also occur prior to initiation of cancer treatment, that is, before start of systemic or radiotherapy, reporting prevalence rates ranging from 14% to 24% [5, 9]. Pretreatment fatigue may be caused by the tumor itself, by tumor surgery or the stress and anxiety of being diagnosed. It seems to emerge as a key predictor influencing the level of fatigue after treatment, resulting in increased fatigue severity and burden in the aftermath [4, 10]. Identifying factors that contribute to or co‐occur with pretreatment fatigue is crucial for alleviating this problem at an early stage, as fatigue can lead to poor cancer treatment adherence [8]. Moreover, investigation of pretreatment fatigue enables identification of risk factors or potential mechanisms that are independent of causal pathways related to therapy. However, compared to research on fatigue during or after cancer treatment, pretreatment fatigue has been less studied with still inconclusive evidence [4, 9, 10, 11, 12, 13].

Some sociodemographic factors have been associated with higher pretreatment fatigue, for example female sex, younger age and lower social support [12, 13]. Inconclusive associations have been found for education level, either showing no association with fatigue [4, 10] or suggesting that lower education is associated with higher fatigue [12]. Besides, higher body mass index (BMI) has been found to be associated with higher fatigue [14], but also showed no association with fatigue levels in other studies [10, 12].

Limited knowledge exists regarding the associations of pretreatment fatigue with other patient‐reported outcomes (PROs). Goedendorp and her colleagues were one of the first to assess pretreatment fatigue in cancer patients and found depressed mood, impaired sleep quality and lower physical activity to be associated with higher fatigue levels [9]. Other studies linked higher levels of pretreatment stress to higher fatigue [15]. Anxiety has been shown to predict fatigue during cancer treatment [16], but the relation to pretreatment fatigue is less clear [9]. Depressive symptoms showed consistently high correlations with increased fatigue levels, before and during cancer treatment [17, 18]. One reason for this might be that fatigue and depressive symptoms share common behavior patterns, for example, tiredness and feeling exhausted. Therefore, it is not surprising that depressive symptoms have been found to be associated with higher levels of fatigue, although causal mechanisms are not yet fully understood and fatigue and depression do not necessarily go hand in hand [18].

Overall, a complete picture of fatigue and its associations with other burdens before start of cancer treatment is lacking. In addition, it remains unclear to what extent pretreatment fatigue in cancer patients differs from fatigue in the general population and thus, might be caused by cancer‐related issues. Identifying factors that contribute to pretreatment fatigue could provide insights into the underlying mechanisms and help to better understand adjustment difficulties depending on patients' individual situations [12]. This is particularly important to provide appropriate support to affected and high‐risk patients and to reduce the potential negative impact of fatigue on patients' quality of life and cancer treatment adherence [10].

To fill the research gaps mentioned above, the aims of this study were as follows: First, we wanted to find out whether patients already suffer from clinically relevant fatigue before the start of treatment and whether their burden was significantly higher compared to a healthy sample. Secondly, we were interested in how patients with clinically relevant pretreatment fatigue were characterized in terms of sociodemographic factors. Taking these sociodemographic factors into account, we then, thirdly, examined the associations between patients with and without clinically relevant fatigue on various psychological factors.

## 2 Methods

### 2.1 Participants

The COGNIFiT study (**COGNI**tive **F**unction **i**n patients with gynecological **T**umors) is a comparative longitudinal study with three measurement timepoints to examine the impact of cancer therapy on cognitive function. Participants were systematically screened and recruited at the National Center of Tumor Diseases (NCT) Heidelberg between November 2018 and March 2023. Eligibility criteria for patients were as follows: they were female, older than 18 years, able to speak and read German as well as be diagnosed with breast cancer or another gynecological tumor. Baseline assessments took place before start of systemic therapy (e.g., chemotherapy, immune therapy, endocrine therapy) and/or radiotherapy. Exclusion criteria were a diagnosis of a psychological, neurological or neurodegenerative illness and having undergone a cancer therapy within the last 2 years (except surgery). Overall, 243 patients were enrolled in the study after providing informed consent. Sample size calculation was based on the primary objective of the study to test for differences in cognitive impairments between different treatments.

To explore whether repetition of neurocognitive testing had any impact on the test results, healthy control participants were recruited via flyer and online advertisement in general practitioners' clinics around Heidelberg, Germany. They were required to meet the same criteria as patients except for having been diagnosed with a breast/gynecological tumor.

However, a comparison between patients and healthy controls was not the primary objective of the study and, accordingly, no power analysis was conducted for this. In the present analyses, we considered the baseline data, which were all assessed before start of systemic cancer treatment or radiotherapy.

The CogniFit study (registered via Deutsches Register Klinischer Studien (DRKS), trial number DRKS00015757) was conducted in accordance with the ethical standards of the Helsinki Declaration. The study was approved by the Ethic Committee of the Medical Faculty of the University of Heidelberg (S‐489/2018).

### 2.2 Assessments

Fatigue was assessed using the EORTC QLQ‐FA12 (FA12). The FA12 has 12‐items with responses on a 4‐point Likert scale. The total fatigue score, and the three subdimensions comprising physical, emotional and cognitive fatigue were calculated and transformed to a scale of 0%–100% according to the scoring manual, with higher scores indicating greater levels of fatigue [19].

Depressive symptoms were measured with the revised 20‐item Center for Epidemiologic Studies Depression Scale (CESD‐R) [20, 21]. Higher scores indicate higher symptom burden. Total questionnaire scores range from 0 (no symptoms) to 80 (maximum symptoms). The frequently used cut‐score of 16 was used to identify possibly depressed patients.

Anxiety was measured using the State Trait Anxiety Inventory (STAI), comprising 20 items [22]. To ensure comparability with other test values, the raw test values were converted into a 0%–100% scale and missing items were imputed [23].

The Pittsburg Sleep Quality Index (PSQI) consists of 19 items asking about sleep quality and sleep disturbances during the past month [24]. Items are divided into 7 subscales, of which the sleep quality subscale was used for the analyses as we considered impaired sleep quality the most relevant variable of the questionnaire with regard to pretreatment fatigue. Higher values on the 0–3 scale represent worse sleep quality.

The EORTC QLQ‐C30 (C30) consists of 30 items divided into five function subscales (physical, role, emotional, cognitive and social), nine symptom subscales/items and a global health subscale [25]. Except the latter, all scales are answered on a 4‐point Likert scale and transformed into a 0%–100% score according to the scoring manual. Patients with clinically relevant functional impairment or fatigue were identified using thresholds of clinical importance (TCI) from previous literature. The TCIs are based on three aspects of patients burden, that is, limitations in everyday life, requiring help or care, or worrying because of symptoms. As interpretation of the 0%–100% scores of the C30 can be challenging, this approach facilitates the interpretation of results by combining significant results with an estimation of their clinical relevance [26].

### 2.3 Statistical Analyses

Data analyses were performed using IBM SPSS statistics version 29 and results were considered statistically significant if *p* was < 0.05. First, descriptive analyses for the PROs were performed separately for patients and healthy participants and differences between the two groups were tested using hierarchical regression analyses with bootstrapping. Bootstrapping was used as a robust statistical method to estimate the standard errors and the confidence intervals for each parameter irrespective of the underlying distribution of the variables.

Applying the TCI for the C30 fatigue score, patients were then divided into two groups: patients with clinically relevant fatigue (C30‐fatigue > 39) and patients without (C30‐fatigue ≤ 39) [26]. To estimate the associations between different sociodemographic variables and clinically relevant fatigue a multiple logistic regression analysis was calculated. Age and social support were entered in the model as continuous variables, whereas education (four levels), BMI (three levels; according to the World Health Organization's (WHO) BMI classification) and the statement if participants exercised in the year before diagnosis (yes/no) were entered as categorial variables. To estimate associations between PROs and fatigue, further logistic regression analyses were conducted. These models were adjusted for the sociodemographic variables that significantly contributed to the association with clinically relevant fatigue. Using only complete cases for regression analyses was considered appropriate as the number of missing data ranged from 0.44% to 2.63% of the study population [27].

## 3 Results

Of the 243 patients enrolled, 236 were diagnosed with breast cancer. The remaining seven patients were diagnosed with other gynecological tumors and were therefore not included in the analyses. Of the 236 breast cancer patients, four did not provide any data on fatigue at all. Therefore, a total of 232 patients were included in the initial statistical analyses. A further four patients did not answer the C30 fatigue questions, so the logistic regressions based on the C30 TCI for fatigue included 228 patients.

The patient sample consisted of 98.7% women who were diagnosed with invasive breast cancer and 1.3% with ductal carcinoma in situ (DCIS). Their mean age was *M* = 55.6 years (SD = 11.6). More than one third (35.8%) of the patients had an academic education, that is, a university degree. Further 21.6% had a high school degree (referring to the German “(Fach‐)Abitur” or a degree of a specialized school, that is, entitling access to universities), while less than half of the patients (42.7%) had a lower qualification than this. Most of the patients (85.4%) lived together with at least one person. The 41 healthy women were slightly younger (*M* = 49.3, SD = 9.8) and better educated compared to the patient sample, as 51.6% had an academic education and only ten women (24.4%) didn't have a high school degree. 40% of this population lived alone. Further sociodemographic characteristics of the study population are depicted in Table 1.

**TABLE 1:** Sociodemographic characteristics of the study population.

	Patients (*N* = 232)	Healthy participants (*N* = 41)
Mean age (SD)	55.6 (11.6)	49.3 (9.8)
Breast cancer type	*N* (%)	*N* (%)
Invasive	229 (98.7%)	
In situ	3 (1.3%)	
Surgery before treatment	*N* (%)	
Yes	226 (97.4%)	
No	6 (2.6%)	
Educational level	*N* (%)	*N* (%)
Academic	83 (35.8%)	23 (56.1%)
Higher education	50 (21.6%)	8 (19.5%)
Moderate education	73 (31.5%)	9 (22.0%)
Basic or no education	26 (11.2%)	1 (2.4%)
BMI in kg/m^2^: Mean (SD)	25.7 (5.4)	24.5 (4.4)
Underweight (< 18.5)	7 (3.0%)	—
Normal (≥ 18.5 and < 25)	122 (52.6%)	26 (63.4%)
Overweight (≥ 25 and < 30)	63 (27.2%)	11 (26.8%)
Obese (≥ 30)	40 (17.2%)	4 (9.7%)
Living alone (household)		
Yes	33 (14.6%)	16 (40%)
No	193 (85.4%)	24 (60%)

*Note:* Regarding living situation, six patients and one healthy participant did not answer the question.

Table 2 displays the distributions of pretreatment fatigue and PROs in patients and healthy participants and the mean differences adjusted for age, BMI and education level. Patients had significantly higher scores on the FA12 total fatigue score (adjusted mean difference = 9.99, 95% confidence interval (CI) [3.83, 15.76]) and on the subscales physical fatigue (12.77, 95% CI [3.94; 21.12]) and emotional fatigue (10.52, 95% CI [2.92; 17.60]), as well as a higher C30 fatigue score (15.96, 95% CI [6.52, 24.64]). Almost half of the patients (48.7%), compared to 17.1% of healthy participants, scored higher than the TCI of 39 on the C30 fatigue symptom subscale, indicating clinically relevant fatigue. Regarding the function scales of the C30, patients reported significantly higher impairment on the subscales physical, role, social, and emotional function (all *p* < 0.01), as well as a worse global health status. Besides, patients reported significantly more depressive symptoms compared to healthy participants (adjusted mean difference = 9.41, 95% CI [4.97, 13.74]), with 48.7% of patients (compared to 22% of healthy participants) scoring higher than the clinically relevant cut‐score of the CESD‐R.

**TABLE 2:** Distributions of patient‐reported outcomes and adjusted mean differences between patients and healthy controls.

Dependent variable	Patients mean (SD)	Patients scoring worse than cut‐score/ TCI^a^	Mean (SD) controls	Controls scoring worse than cut‐score/ TCI	Adjusted mean difference [95% CI]	*T‐*test	*p*
EORTC QLQ‐FA12							
Total fatigue	28.42 (22.82)	n.a.	16.73 (15.65)	n.a.	**9** **.99 [3.83, 15.76]**	**2.62**	**0.009**
Physical fatigue	39.94 (28.30)	n.a.	24.23 (21.02)	n.a.	**12.77 [3.94, 21.12]**	**2.71**	**0.007**
Emotional fatigue	25.60 (28.78)	n.a.	15.18 (19.04)	n.a.	**10.52 [2.92, 17.60]**	**2.17**	**0.031**
Cognitive fatigue	11.58 (18.80)	n.a.	5.69 (10.28)	n.a.	5.85 [1.45, 9.99]	1.89	0.060
EORTC QLQ‐C30							
Fatigue	40.84 (27.44)	48.7%	22.49 (24.28)	17.1%	**15.96 [6.52, 24.64]**	**3.45**	**< 0.001**
Physical function	80.56 (19.97)	47.4%	92.85 (13.20)	12.2%	**−8.70 [−13.08, −3.85]**	**−2.74**	**0.007**
Role function	62.19 (31.83)	39.6%	92.28 (15.85)	4.9%	**−28.98 [−35.43, −22.29]**	**−5.54**	**< 0.001**
Social function	72.66 (30.18)	26.3%	94.17 (13.89)	5.0%	**−21.06 [−27.24, −14.86]**	**−4.21**	**< 0.001**
Cognitive function	77.68 (24.51)	37.4%	85.77 (19.21)	24.4%	−6.28 [−12.91, 0.77]	−1.52	0.129
Emotional function	55.62 (26.35)	65.5%	71.75 (20.49)	51.2%	**−16.54 [−24.07, −9.16]**	**−3.68**	**< 0.001**
Global health status	61.60 (20.76)	n.a.	81.10 (12.91)	n.a.	**−16.75 [−21.93, −11.49]**	**−4.91**	**< 0.001**
Depression (CESD‐R)							
Total score	20.54 (19.07)	48.7%	9.31 (11.14)	22%	**9.41 [4.97, 13.74]**	**3.01**	**0.003**
Anxiety (STAI)							
Total score %	32.44 (19.56)	n.a.	25.94 (17.48)	n.a.	6.68 [0.44, 12.79]	1.92	0.056
Sleep quality	1.39 (0.73)	n.a.	1.15 (0.53)	n.a.	0.174 [−0.05, 0.38]	1.47	0.149

*Note:* We calculated separate models for all listed dependent variables. All models were adjusted for age, BMI and education. Bootstrapping based on 5000 bootstrap samples was used to calculate the coefficients and the confidence intervals, as residuals were not normally distributed. Significant results (*p* < 0.05) were marked in bold.

Abbreviations: CESD‐R = Center for Epidemiological Studies Depression Scale—Revised, CI = confidence interval, n.a. = not applicable, when no cut‐scores or thresholds of clinical importance were defined for these variables, STAI = State Trait Anxiety Inventory.

^a^ TCI = thresholds of clinical importance for the EORTC QLQ C30 subscales [26]. Cut‐score for the CESD‐R = 16 [20, 21].

With regard to sociodemographic variables (see Table 3), the odds for showing clinically relevant fatigue were higher for patients with no/basic education (OR = 2.87, 95% CI [1.05; 7.84]) or moderate education (OR = 2.79, 95% CI [1.39; 5.60]) compared to those with academic education, for those with lower social support (OR = 0.96; 95% CI [0.94; 0.99]) compared to those with higher social support, and also tended to be higher for younger compared to older patients (OR = 0.98; 95% CI [0.95; 1.00]). Obese patients (BMI ≥ 30) had higher odds for showing clinically relevant fatigue compared to patients with a normal BMI (OR = 2.56, 95% CI [1.14; 5.72]).

**TABLE 3:** Logistic regression analysis to estimate associations of sociodemographic variables with clinically relevant fatigue in 228 breast cancer patients before treatment.

Dependent variable: clinically relevant fatigue (yes vs. no)^a^	OR [95% CI]	*p*
Age	0.98 [0.95; 1.00]	0.054
Social support	0.96 [0.94; 0.99]	0.001
Exercise before diagnosis		
Yes	Ref	
No	1.07 [0.57; 2.02]	0.835
Educational level		
Academic	Ref	
Higher education	1.78 [0.83; 3.84]	0.140
Moderate education	2.79 [1.39; 5.60]	0.004
No or basic education	2.87 [1.05; 7.84]	0.039
BMI		
Normal^b^ (BMI < 25)	Ref	
Overweight (BMI ≥ 25 and < 30)	0.77 [0.39; 1.49]	0.402
Obese (BMI ≥ 30)	2.56 [1.14; 5.72]	0.022

*Note:* All listed variables were included simultaneously. *R* ^2^ (Nagelkerke) = 0.172.

Abbreviations: BMI = body mass index, CI = confidence interval (based on 1000 bootstrap samples), OR = odds ratio.

^a^ Based on the threshold of clinical importance for the EORTC QLQ‐C30 subscales [26].

^b^ The seven patients with BMI ranging between 16 and 18 were also included in this category for reasons of parsimony.

**FIGURE 1:**
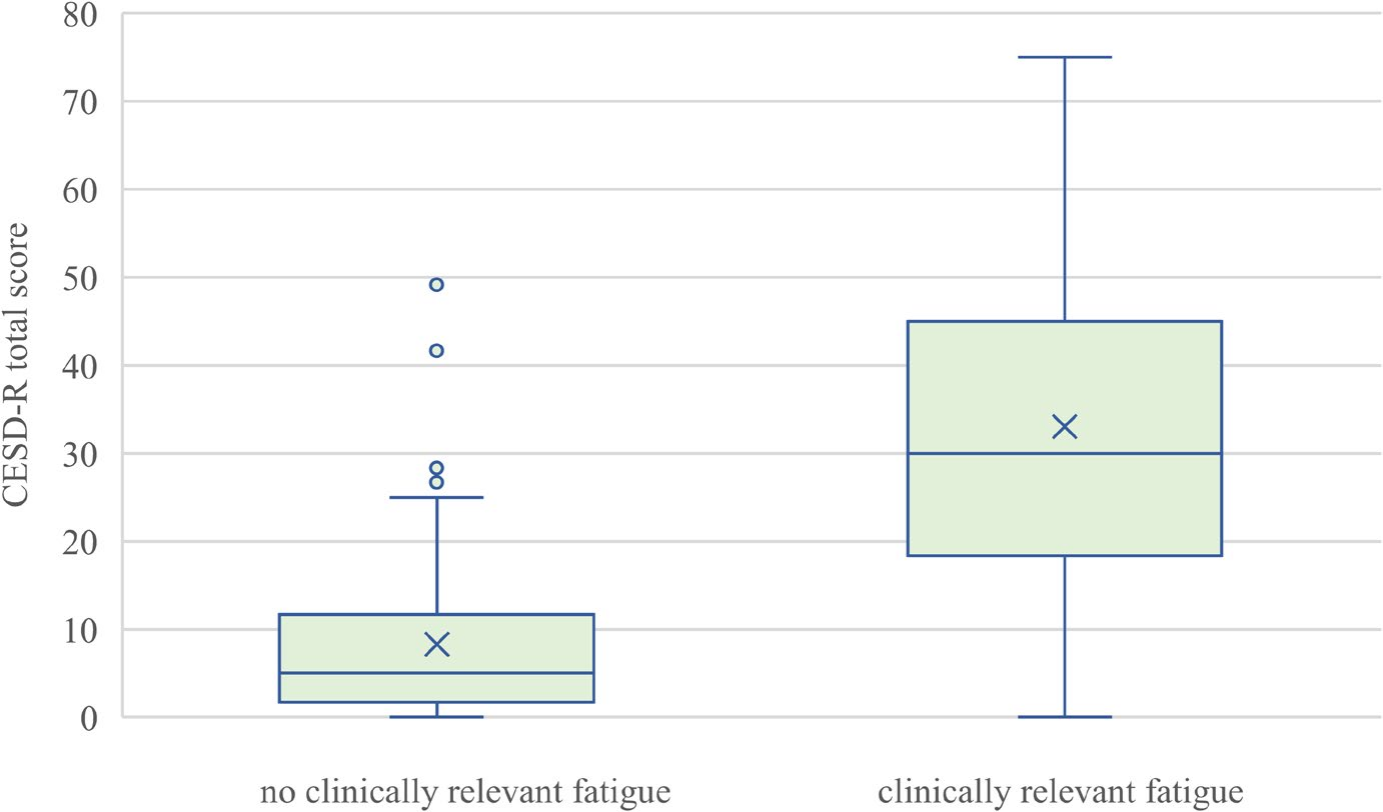
Distribution of depressive symptoms between patients with and without clinically relevant fatigue. CESD‐R = Center for Epidemiologic Studies Depression Scale—Revised.

**FIGURE 2:**
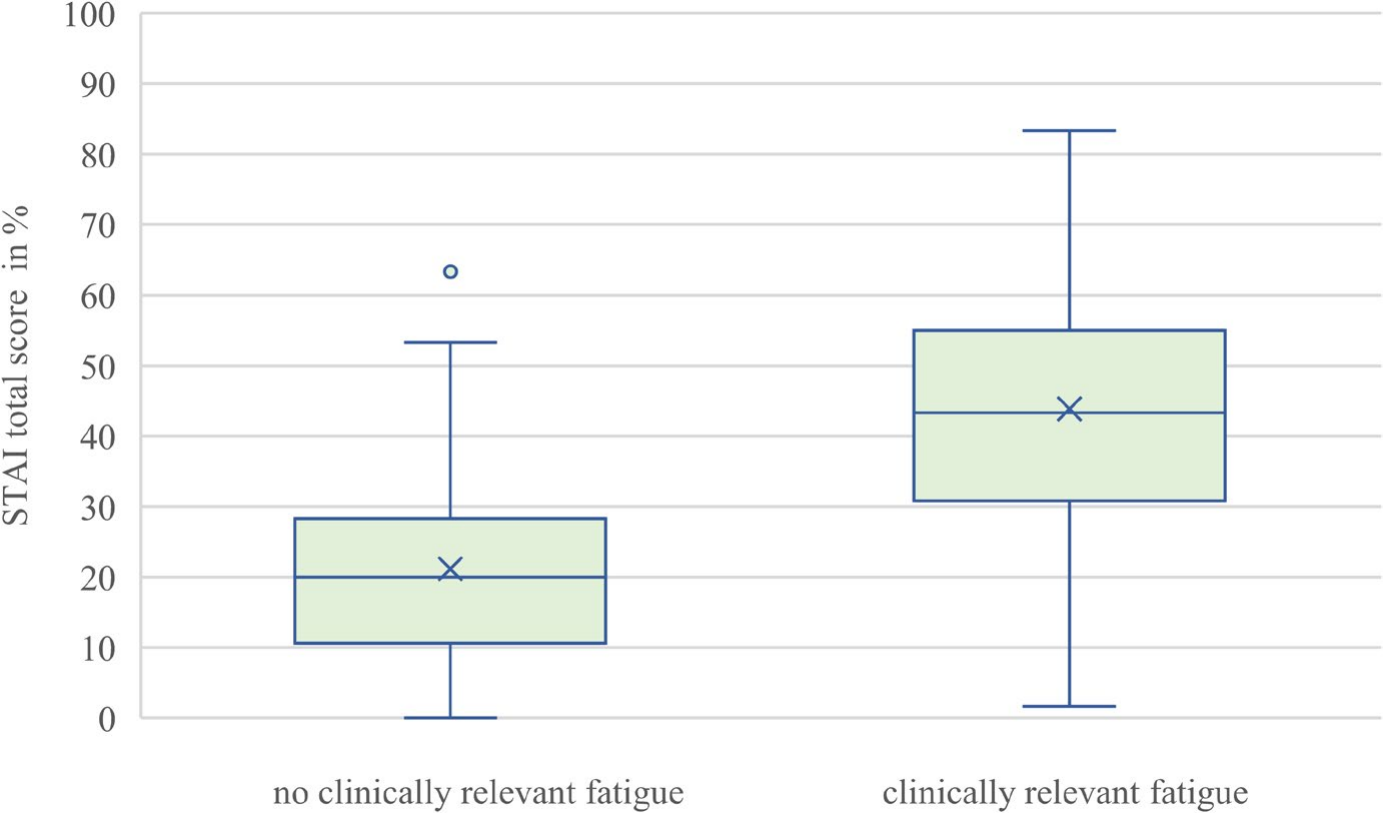
Distribution of anxiety symptoms between patients with and without clinically relevant fatigue. STAI = State Trait Anxiety Inventory.

**FIGURE 3:**
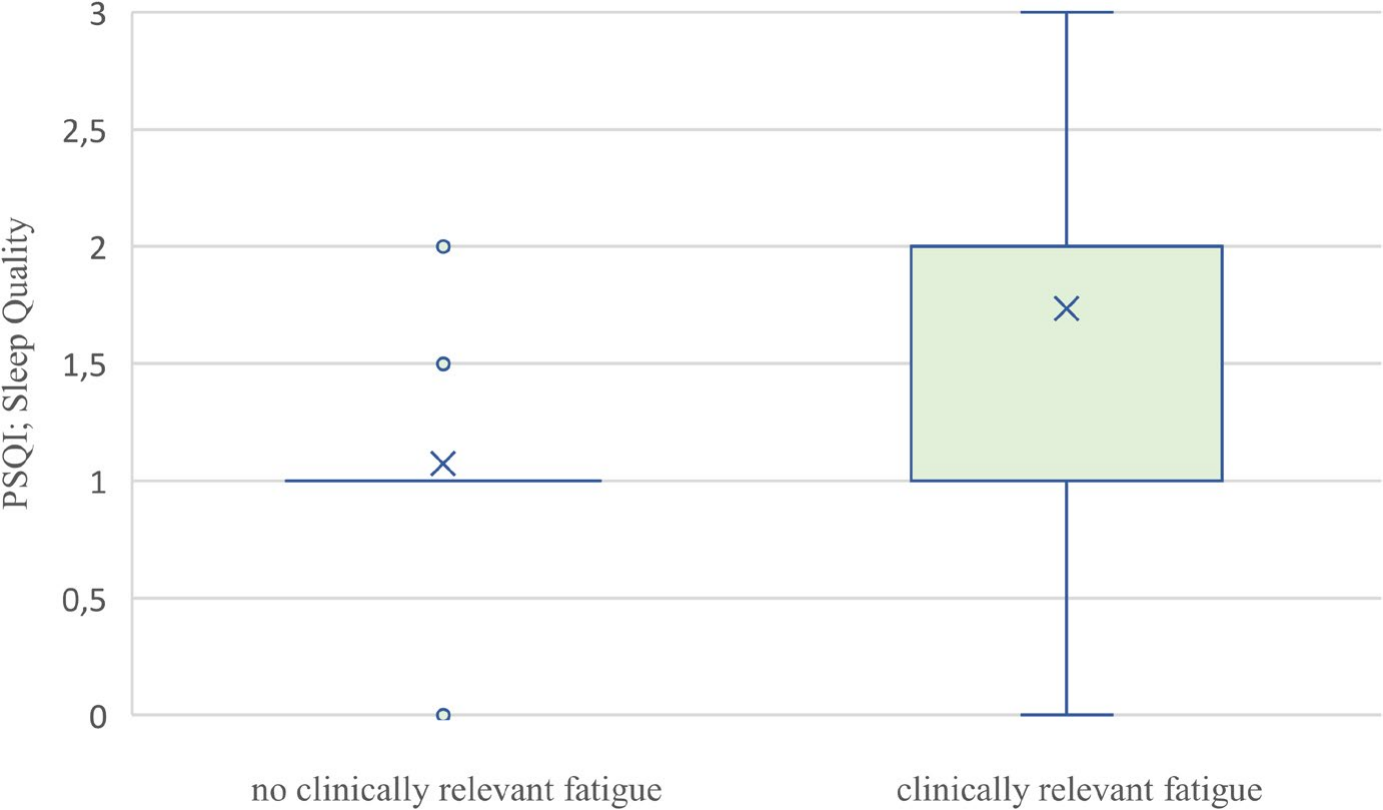
Distribution of impaired sleep quality between patients with and without clinically relevant fatigue. PSQI = Pittsburg Sleep Quality Index; subscale Sleep Quality.

Table 4 shows the results of multiple logistic regression models to estimate associations of other PROs with clinically relevant fatigue in patients. Clinically relevant fatigue was associated with higher symptom burden regarding depression (OR = 1.17, 95% CI [1.12; 1.22]) and anxiety (OR = 1.10, 95% CI [1.07; 1.13]), poorer sleep quality (OR = 5.18, 95% CI [3.00; 8.93]), a worse global health status (OR = 0.90; 95% CI [0.88; 0.93]), as well as lower scores in all five function scales of the C30 (all *p* < 0.001). Figures 1, 2, 3 illustrate the distribution of symptom burden between patients with and without clinically relevant fatigue. Figures showing lower functioning in social, emotional, physical, role, and cognitive domains and global health status in patients with clinically relevant fatigue compared with those without are also provided in the Appendix (Figures S4a and S4b).

**TABLE 4:** Separate logistic regression models to estimate associations of patient‐reported outcomes with clinically relevant fatigue in 228 breast cancer patients before treatment.

Dependent variable: clinically relevant fatigue (yes vs. no)	OR [95% CI]	*p*	Pseudo *R* ^2^ (Nagelkerke)
Depression (total score)	1.17 [1.12; 1.22]	< 0.001	0.63
Anxiety (total score)	1.10 [1.07; 1.13]	< 0.001	0.51
Sleep quality^a^	5.18 [3.00; 8.93]	< 0.001	0.36
EORTC QLQ C30			
Physical function	0.91 [0.89; 0.94]	< 0.001	0.48
Role function	0.95 [0.93; 0.96]	< 0.001	0.52
Social function	0.94 [0.93; 0.96]	< 0.001	0.51
Cognitive function	0.93 [0.91; 0.95]	< 0.001	0.48
Emotional function	0.93 [0.91; 0.95]	< 0.001	0.58
Global health status	0.90 [0.88; 0.93]	< 0.001	0.57

*Note:* Each line represents a separate model, adjusted for age, social support, BMI and education. Depressive symptoms were measured with the Center for Epidemiologic Studies Depression Scale – Revised (CESD‐R, ranging from 0 to 80), anxiety was measured with the State Trait Anxiety Inventory (STAI, ranging from 0% to 100%); sleep quality was measured with a subscale (ranging from 0 to 3) of the Pittsburg Sleep Quality Index (PSQI).

Abbreviations: CI = Confidence Interval (based on 1000 bootstrap samples), OR = Odds Ratio.

^a^ Higher scores on this scale indicate poorer sleep quality.

## 4 Discussion

In patients, we found significantly higher scores for physical, emotional and total fatigue as well as for the fatigue subscale score of the C30 compared to healthy participants, even after adjusting for sociodemographic factors. These results point to a higher fatigue burden in patients already at the beginning of cancer treatment.

Our patient sample showed lower fatigue scores in all FA12 dimensions as well as in the total score compared to patients already under treatment [28]. Nevertheless, almost half of our breast cancer patients scored worse than the TCI for clinically relevant fatigue even before starting therapy, indicating supportive care needs. More precisely, scoring higher than the TCI represents a significant limitation to the patient's daily life due to the perceived symptom burden, causes worry to patients or their families, or requires help or care [26]. As it is well‐known that fatigue symptoms tend to worsen with treatment, these results may only indicate the beginning of the fatigue burden, emphasizing the need to educate patients and their relatives about fatigue at an early stage.

Our results support findings from previous literature, which found that lower age and low social support are associated with higher fatigue [12, 29]. With regard to the inconclusive findings to date on whether education and BMI play a role in pretreatment fatigue [4, 10, 12, 14], our data suggest that they do. These findings may guide health care professionals in identifying patients at high risk of developing fatigue prior to treatment. For example, as BMI is a modifiable factor, advice on diet and physical activity could not only reduce weight, but also prevent the development or worsening of fatigue [30]. As lower education also appears to be a risk factor for fatigue, providing adequate and easily understandable information about cancer and its side effects could be an important pillar of supportive cancer care.

In the patient group, higher depression and anxiety scores and poorer sleep quality seemed to get along with an increased likelihood of scoring above the TCI of fatigue. In light of previous inconclusive findings, our results may suggest that anxiety already plays a significant role in pretreatment fatigue levels. This assumption is underlined by a recent systematic review by Cheng and colleagues, who also found that anxiety and depression were associated with higher odds of experiencing increased fatigue [31]. Other studies have reported similar findings, emphasizing that psychological factors in general play an important role in pretreatment fatigue [9, 10].

The significantly higher depression score in patients compared to healthy participants leads to the interpretation that a cancer diagnosis can cause tremendous emotional distress [10]. The fact that we only included individuals with no pre‐diagnosed mental illness in our study makes it even more likely that the cancer diagnosis/surgery was the cause of the increased symptom burden. Using the classical cut score of 16 [20, 21], almost half of the patients reported a clinically relevant symptom burden of depression. This is a considerably high number of people who may be depressed. However, this number may be overestimated, as research has shown that the cut score of 16 may lack specificity in some study populations, and 22% of our healthy participants scored above this score [32]. Nevertheless, pretreatment signs of depression should be taken seriously, as they are a strong predictor of poorer well‐being during and after treatment [33].

Regarding the mean scores of the C30 function subscales, we found slightly lower, but still comparable values in our patient sample compared to the reference values of early breast cancer patients prior to treatment [34]. The mean scores of the healthy sample were comparable to the German norm values [35]. Patients with clinically relevant fatigue scored worse on all function subscales than those without, pointing to a potentially significant negative impact of pretreatment fatigue on other life domains and the global health status. Underlying common causes, such as increased distress levels, may account for this association.

Overall, our results show that fatigue does not occur alone but mostly clusters with other psychological and functional factors [3, 31]. Almost half of breast cancer patients in our study experienced clinically relevant fatigue already before start of treatment. Thus, their fatigue was caused by other factors than radio‐ or systemic therapies. Although we cannot point to any causal relationships here, the data suggest that the TCI of fatigue can reliably distinguish between patients regarding overall symptom burden. It might therefore be promising if future research investigates TCIs for the FA12 subscales to provide a more nuanced picture of pretreatment fatigue burden in cancer patients.

### 4.1 Clinical Implications

Our findings highlight the importance of screening patients for fatigue even before they start cancer treatment and providing them with comprehensive information about this distressing side effect [10, 30]. More specifically, the provision of easy‐to‐understand educational information appears not only to reduce symptoms, but also to facilitate positive outcomes and is in line with international guideline recommendations [1, 30, 36, 37]. Appropriate information should include a description of symptoms and effective treatment options, such as physical activity or mind–body exercises [1, 30].

As social support is also known to be a great resource for cancer patients and to have a positive influence on treatment (adherence), providing support to socially disadvantaged people seems particularly important. Here, for example, cognitive behavioral therapy to help patients address emotional distress and cope with their diagnosis has been shown to be effective [38].

As our results also suggest that psychological mechanisms appear to be triggered by the diagnosis, psychosocial treatments/interventions should be offered to affected patients prior to starting treatment [39]. This may not only help to manage psychological distress but may also have a positive effect on fatigue levels [30, 40].

In summary, the provision of educational, emotional and psychosocial support, including cognitive behavioral therapy, may address patients' concerns and needs prior to treatment initiation, potentially reducing psychological distress, improving sleep quality and reducing fatigue. If time constraints in routine clinical practice require prioritization, it may be possible to focus on high‐risk patients as characterized here.

### 4.2 Study Limitations

Some limitations of the study need to be considered. First, the study population was limited to patients diagnosed with breast cancer. Therefore, it remains an open question whether the findings can be extrapolated to other cancer entities. Given the different sample sizes and characteristics of patients and healthy controls, the statistical differences observed in the results must be interpreted with caution. In addition, as we only examined patient‐reported burden cross‐sectionally, causal interpretations of our findings cannot be made and adjustment for pre‐diagnosis fatigue was not possible. All data analyzed were self‐reported; however, they were assessed using validated questionnaires and showed marked differences in psychological symptom burden between clinically relevant fatigued and non‐fatigued patients.

## 5 Conclusions

To ensure an adequate fatigue management, fatigue screening, patient‐centered education on fatigue and psychosocial support may be needed already from the time of cancer diagnosis. Providing coping and symptom management strategies prior to starting cancer treatment may be an important step in preventing further exacerbation or chronicity of fatigue.

## Author Contributions

**Patricia Blickle:** formal analysis (lead), writing – original draft (lead). **Martina E. Schmidt:** conceptualization (equal), formal analysis (supporting), project administration (equal), writing – original draft (supporting). **Karen Steindorf:** conceptualization (equal), investigation (equal), project administration (equal), writing – original draft (supporting).

## Conflicts of Interest

Martina E. Schmidt and Patricia Blickle declare no conflicts of interest. Karen Steindorf received honararies and travel refunds for talks with some relation to the context of the manuscript from the companies Takeda and Adviva medical equipment.

## Supporting information


Figure S1.

## Data Availability

Data can be made available to scientific cooperation partners upon reasonable request.

## References

[1] A. M. Berger , K. Mooney , and A. Alvarez‐Perez , et al., National Comprehensive Cancer Network , “NCCN Clinical Practice Guidelines in Oncology: Cancer‐Related Fatigue. Version 2.2023,” Journal of National Comprehensive Cancer Network 13, no. 8 (2015): 1012–1039, 10.6004/jnccn.2015.0122. PMC5499710 26285247

[2] T. Islam , M. Dahlui , H. A. Majid , A. M. Nahar , N. A. Mohd Taib , and T. T. Su , “Factors Associated With Return to Work of Breast Cancer Survivors: A Systematic Review,” BioMed Central Public Health 14 (2014): S8. 10.1186/1471-2458-14-S3-S8 PMC4251139 25437351

[3] J. E. Bower , “Cancer‐Related Fatigue–Mechanisms, Risk Factors, and Treatments,” Nature Reviews. Clinical Oncology 11 (2014): 597–609. 10.1038/nrclinonc.2014.127 PMC4664449 25113839

[4] M. M. Goedendorp , M. F. Gielissen , C. A. Verhagen , and G. Bleijenberg , “Development of Fatigue in Cancer Survivors: A Prospective Follow‐Up Study From Diagnosis Into the Year After Treatment,” Journal of Pain and Symptom Management 45 (2013): 213–222. 22926087 10.1016/j.jpainsymman.2012.02.009

[5] C. G. Brownstein , R. Twomey , J. Temesi , et al., “Physiological and Psychosocial Correlates of Cancer‐Related Fatigue,” Journal of Cancer Survivorship 16 (2022): 1339–1354. 34609702 10.1007/s11764-021-01115-6

[6] M. E. Schmidt , S. Goldschmidt , S. Hermann , and K. Steindorf , “Late Effects, Long‐Term Problems and Unmet Needs of Cancer Survivors,” International Journal of Cancer 151 (2022): 1280–1290. 35657637 10.1002/ijc.34152

[7] M. Al Maqbali , M. Al Sinani , Z. Al Naamani , K. Al Badi , and M. I. Tanash , “Prevalence of Fatigue in Patients With Cancer: A Systematic Review and Meta‐Analysis,” Journal of Pain and Symptom Management 61 (2021): 167–189.e14. 32768552 10.1016/j.jpainsymman.2020.07.037

[8] M. Hofman , J. L. Ryan , C. D. Figueroa‐Moseley , P. Jean‐Pierre , and G. R. Morrow , “Cancer‐Related Fatigue: The Scale of the Problem,” Oncologist 12 (2007): 4–10. 17573451 10.1634/theoncologist.12-S1-4

[9] M. M. Goedendorp , M. F. Gielissen , C. A. Verhagen , M. E. Peters , and G. Bleijenberg , “Severe Fatigue and Related Factors in Cancer Patients Before the Initiation of Treatment,” British Journal of Cancer 99 (2008): 1408–1414. 18941462 10.1038/sj.bjc.6604739 PMC2579682

[10] M. M. Pertl , D. Hevey , S. Collier , K. Lambe , and A. M. O'Dwyer , “Predictors of Fatigue in Cancer Patients Before and After Chemotherapy,” Journal of Health Psychology 19 (2014): 699–710. 23479299 10.1177/1359105313477675

[11] D. Goldstein , B. K. Bennett , K. Webber , et al., “Cancer‐Related Fatigue in Women With Breast Cancer: Outcomes of a 5‐Year Prospective Cohort Study,” Journal of Clinical Oncology 30 (2012): 1805–1812. 22508807 10.1200/JCO.2011.34.6148

[12] J. E. Bower , A. Asher , D. Garet , et al., “Testing a Biobehavioral Model of Fatigue Before Adjuvant Therapy in Women With Breast Cancer,” Cancer 125 (2019): 633–641. 30561795 10.1002/cncr.31827 PMC6373488

[13] J. C. Rosas , M. E. Aguado‐Barrera , D. Azria , et al., “(Pre)treatment Risk Factors for Late Fatigue and Fatigue Trajectories Following Radiotherapy for Breast Cancer,” International Journal of Cancer 153 (2023): 1579–1591. 37403702 10.1002/ijc.34640

[14] L. H. Gerber , N. Stout , C. McGarvey , et al., “Factors Predicting Clinically Significant Fatigue in Women Following Treatment for Primary Breast Cancer,” Support Care Cancer 19 (2011): 1581–1591. 20835835 10.1007/s00520-010-0986-7 PMC3166607

[15] P. B. Jacobsen , D. M. Hann , L. M. Azzarello , J. Horton , L. Balducci , and G. H. Lyman , “Fatigue in Women Receiving Adjuvant Chemotherapy for Breast Cancer: Characteristics, Course, and Correlates,” Journal of Pain and Symptom Management 18 (1999): 233–242. 10534963 10.1016/s0885-3924(99)00082-2

[16] A. Hughes , S. Suleman , K. A. Rimes , J. Marsden , and T. Chalder , “Cancer‐Related Fatigue and Functional Impairment—Towards an Understanding of Cognitive and Behavioural Factors,” Journal of Psychosomatic Research 134 (2020): 110127. 32428784 10.1016/j.jpsychores.2020.110127

[17] K. Susanne , F. Michael , S. Thomas , E. Peter , and H. Andreas , “Predictors of Fatigue in Cancer Patients: A Longitudinal Study,” Support Care Cancer 27 (2019): 3463–3471. 30680616 10.1007/s00520-019-4660-4

[18] Y. Wang , L. Tian , X. Liu , et al., “Multidimensional Predictors of Cancer‐Related Fatigue Based on the Predisposing, Precipitating, and Perpetuating (3P) Model: A Systematic Review,” Cancers (Basel) 15 (2023): 15. 10.3390/cancers15245879 PMC10741552 38136423

[19] J. Weis , K. A. Tomaszewski , E. Hammerlid , et al., EORTC Quality of Life Group , “International Psychometric Validation of an EORTC Quality of Life Module Measuring Cancer Related Fatigue (EORTC QLQ‐FA12),” Journal of the National Cancer Institute 109, no. 5 (2017), 10.1093/jnci/djw273. 28376231

[20] L. S. Radloff , “The CES‐D Scale: A Self‐Report Depression Scale for Research in the General Population,” Applied Psychological Measurement 1 (1977): 385–401.

[21] W. W. Eaton , C. Smith , M. Ybarra , M. Carles , and A. Tien , “Center for Epidemiologic Studies Depression Scale: Review and Revision (CESD and CESD‐R),” Use of Psychological Testing for Treatment Planning and Outcomes Assessment: Instruments for Adults 3 (2004): 363–377.

[22] C. D. Spielberger , “State‐Trait Anxiety Inventory for Adults,” 1983.

[23] J. (Hg.). Grimm , State‐Trait‐Anxiety Inventory Nach Spielberger. Deutsche Lang‐und Kurzversion (Methodenforum der Universität Wien, MF‐Working Paper, 2009/02).

[24] D. J. Buysse , C. F. Reynolds, III , T. H. Monk , S. R. Berman , and D. J. Kupfer , “The Pittsburgh Sleep Quality Index: A New Instrument for Psychiatric Practice and Research,” Psychiatry Research 28 (1989): 193–213. 2748771 10.1016/0165-1781(89)90047-4

[25] P. Fayers and A. Bottomley , “Quality of Life Research Within the EORTC‐The EORTC QLQ‐C30. European Organisation for Research and Treatment of Cancer,” European Journal of Cancer 38, no. Suppl 4 (2002): S125–S133. 11858978 10.1016/s0959-8049(01)00448-8

[26] J. M. Giesinger , F. L. C. Loth , N. K. Aaronson , et al., “Thresholds for Clinical Importance Were Established to Improve Interpretation of the EORTC QLQ‐C30 in Clinical Practice and Research,” Journal of Clinical Epidemiology 118 (2020): 1–8. 31639445 10.1016/j.jclinepi.2019.10.003

[27] D. Urban , J. Mayerl , and A. Wahl , “Regressionsanalyse bei fehlenden Variablenwerten (Missing Values): Imputation oder Nicht‐Imputation? Eine Anleitung für die Regressionspraxis mit SPSS,” in Regression Analysis When Variables Have Missing Values: Imputation or no Imputation? A Guide for Practical Regression Analysis With SPSS, 44 (Schriftenreihe des Instituts für Sozialwissenschaften der Universität Stuttgart (SISS), 2016).

[28] S. Kecke , J. Ernst , J. Einenkel , S. Singer , and A. Hinz , “Psychometric Properties of the Fatigue Questionnaire EORTC QLQ‐FA12 in a Sample of Female Cancer Patients,” Journal of Pain and Symptom Management 54 (2017): 922–928. 28807705 10.1016/j.jpainsymman.2017.08.007

[29] O. Husson , F. Mols , L. van de Poll‐Franse , J. de Vries , G. Schep , and M. S. Thong , “Variation in Fatigue Among 6011 (Long‐Term) Cancer Survivors and a Normative Population: A Study From the Population‐Based PROFILES Registry,” Support Care Cancer 23 (2015): 2165–2174. 25556703 10.1007/s00520-014-2577-5

[30] A. Fabi , R. Bhargava , S. Fatigoni , et al., “Cancer‐Related Fatigue: ESMO Clinical Practice Guidelines for Diagnosis and Treatment,” Annals of Oncology 31 (2020): 713–723. 32173483 10.1016/j.annonc.2020.02.016

[31] Z. Cheng , A. Johar , M. Nilsson , A. Schandl , and P. Lagergren , “Cancer‐Related Fatigue Trajectories up to 5 Years After Curative Treatment for Oesophageal Cancer,” British Journal of Cancer 130 (2023): 628–637. 38135716 10.1038/s41416-023-02551-0 PMC10876982

[32] M. M. Weissman , D. Sholomskas , M. Pottenger , B. A. Prusoff , and B. Z. Locke , “Assessing Depressive Symptoms in Five Psychiatric Populations: A Validation Study,” American Journal of Epidemiology 106 (1977): 203–214. 900119 10.1093/oxfordjournals.aje.a112455

[33] L. Liu , L. Fiorentino , L. Natarajan , et al., “Pre‐Treatment Symptom Cluster in Breast Cancer Patients Is Associated With Worse Sleep, Fatigue and Depression During Chemotherapy,” Psycho‐Oncology 18 (2009): 187–194. 18677716 10.1002/pon.1412 PMC2762479

[34] M. M. Karsten , R. Roehle , S. Albers , et al., “Real‐World Reference Scores for EORTC QLQ‐C30 and EORTC QLQ‐BR23 in Early Breast Cancer Patients,” European Journal of Cancer 163 (2022): 128–139. 35066338 10.1016/j.ejca.2021.12.020

[35] R. Schwarz and A. Hinz , “Reference Data for the Quality of Life Questionnaire EORTC QLQ‐C30 in the General German Population,” European Journal of Cancer 37 (2001): 1345–1351. 11435063 10.1016/s0959-8049(00)00447-0

[36] P. Blickle , M. E. Schmidt , and K. Steindorf , “Post‐Traumatic Growth in Cancer Survivors: What is Its Extent and What Are Important Determinants?,” International Journal of Clinical and Health Psychology 24 (2024): 100418. 37867603 10.1016/j.ijchp.2023.100418 PMC10585376

[37] M. E. Schmidt , M. Milzer , C. Weiß , P. Reinke , M. Grapp , and K. Steindorf , “Cancer‐Related Fatigue: Benefits of Information Booklets to Improve Patients' Knowledge and Empowerment,” Support Care Cancer 30 (2022): 4813–4821. 35147759 10.1007/s00520-022-06833-w PMC8853058

[38] A. Guarino , C. Polini , G. Forte , F. Favieri , I. Boncompagni , and M. Casagrande , “The Effectiveness of Psychological Treatments in Women With Breast Cancer: A Systematic Review and Meta‐Analysis,” Journal of Clinical Medicine 9 (2020): 9. 10.3390/jcm9010209 PMC7019270 31940942

[39] S. Schneider , A. Moyer , S. Knapp‐Oliver , S. Sohl , D. Cannella , and V. Targhetta , “Pre‐Intervention Distress Moderates the Efficacy of Psychosocial Treatment for Cancer Patients: A Meta‐Analysis,” Journal of Behavioral Medicine 33 (2010): 1–14. 19784868 10.1007/s10865-009-9227-2 PMC2813921

[40] H. T. Myrhaug , J. A. Mbalilaki , N. K. Lie , T. Hansen , and J. E. Nordvik , “The Effects of Multidisciplinary Psychosocial Interventions on Adult Cancer Patients: A Systematic Review and Meta‐Analysis,” Disability and Rehabilitation 42 (2020): 1062–1070. 30497305 10.1080/09638288.2018.1515265

