# Supplementary material for: Pretreatment Fatigue in Breast Cancer Patients: Comparison With Healthy Controls and Associations With Biopsychosocial Variables
Source: Cancer Med. 2025 Jan 9;14(1):e70404. doi: 10.1002/cam4.70404 (PMC11712150; doi:10.1002/cam4.70404)
Supplement: Supplementary file 1 — Figure S1. [file CAM4-14-e70404-s001.docx]

**Figure 4.** Extent and distribution of scoring of patients with (Figure A1a) and patients
without (Figure A1b) clinically relevant fatigue on the EORTC QLQ-C30 function subscales.

4a


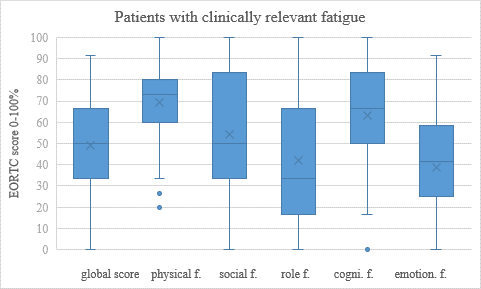


4b


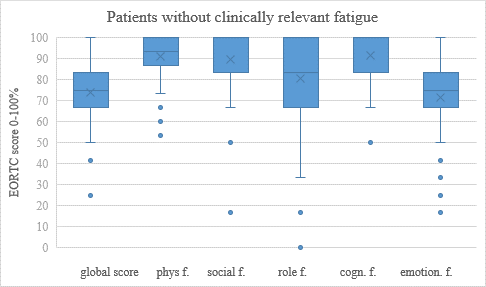


*Note.* Subscales were transformed into a 0-100% scale according to the manual.^19^
phys. = physical, cogni. = cognitive, emotion. = emotional, f. = functioning.
